# AAV delivered lysosome-targeting chimeras mediate sustained antibody depletion *in vivo*

**DOI:** 10.64898/2026.07.05.736665

**Published:** 2026-08-03

**Authors:** Jonathan Lee Yang, Kang Yong Loh, Cindy R. Sandoval Espinoza, Dina Schuster, Karl Deisseroth, Carolyn R. Bertozzi

## Abstract

Immunoglobulins (e.g., IgGs) are critical effectors of the adaptive immune system that when overexpressed or dysregulated can result in autoimmune diseases. Thus, depletion of IgGs can be a promising therapeutic avenue. Here we developed g enetically- e ncoded lysosome targeting c himeras (GELYTACs) that target circulating IgGs for clearance and degradation. The GELYTACs comprised two protein modules derived from insulin-like growth factor 2 (IGF2) and an IgG-binding nanobody, respectively, and mediated clearance of plasma IgG via the lysosomal trafficking receptor IGF2R. To achieve long-lasting IgG depletion, we encoded GELYTACs in an AAV gene therapy vector and established continuous expression in mice. We also developed conditional GELYACs that are activatable with disease-specific proteases or small molecule drugs. This work establishes GELYTACs as a possible therapeutic modality that is deliverable using genetic medicine approaches.

## Full Text Availability

The license terms selected by the author(s) for this preprint version do not permit archiving in PMC. The full text is available from the preprint server.

